# Difference between estimated glomerular filtration rate based on cystatin C versus creatinine and cardiovascular–kidney–metabolic health

**DOI:** 10.3389/fmed.2024.1477343

**Published:** 2025-01-15

**Authors:** Xiaoyan Wu, Wuming Hu, Jian Xu, Jiayi Shen, Li Lin, Jingshuai Zhu, Tiemin Wei, Lingchun Lv

**Affiliations:** ^1^Department of Cardiology, Lishui Central Hospital and the Fifth Affiliated Hospital of Wenzhou Medical University, Lishui, China; ^2^Department of Cardiology, Lishui Hospital, Zhejiang University School of Medicine, Lishui, China

**Keywords:** cystatin C, estimated glomerular filtration rate, serum creatinine, cardiovascular–kidney–metabolic, NHANES

## Abstract

**Background:**

The difference between the estimated glomerular filtration rate (eGFR) calculated from cystatin C and creatinine (eGFRdiff) serves as a biomarker of kidney function impairment. However, the role of eGFRdiff in cardiovascular–kidney–metabolic (CKM) health and its impact on mortality in CKM syndrome patients has not yet been studied.

**Methods:**

This study included 3,622 participants from the National Health and Nutrition Examination Survey (NHANES) conducted between 1999 and 2004. Weighted ordinal logistic regression was used to explore the link between eGFRdiff and CKM health, while weighted Cox regression was used to examine the relationship between eGFRdiff and mortality in CKM syndrome patients. Restricted cubic splines (RCSs) were used to analyze the dose–response relationship.

**Results:**

The common odds ratio (cOR) per 10 mL/min/1.73m^2^ increase in eGFRdiff was 0.86 [95% confidence interval (CI), 0.81 to 0.91]. Compared to the midrange eGFRdiff, the cOR values for the negative and positive eGFRdiff were 1.88 [95% CI, 1.23 to 2.88] and 0.69 [95% CI, 0.58 to 0.83], respectively. During a median follow-up of 201 months, 853 participants died from all causes, while 265 died due to cardiovascular causes. The hazard ratios (HRs) per 10 mL/min/1.73m^2^ increase in eGFRdiff were 0.88 [95% CI, 0.83 to 0.93] for all-cause mortality and 0.90 [95% CI, 0.81 to 1.00] for cardiovascular mortality cases. Compared to the participants with a midrange eGFRdiff, those with negative eGFRdiff had a 48% higher risk of all-cause mortality, while those with positive eGFRdiff had a 30% lower risk. No significant non-linear associations were found in these regression analyses.

**Conclusion:**

Our study found that eGFRdiff is associated with CKM health and stratified mortality risk in CKM syndrome patients.

## Graphical abstract

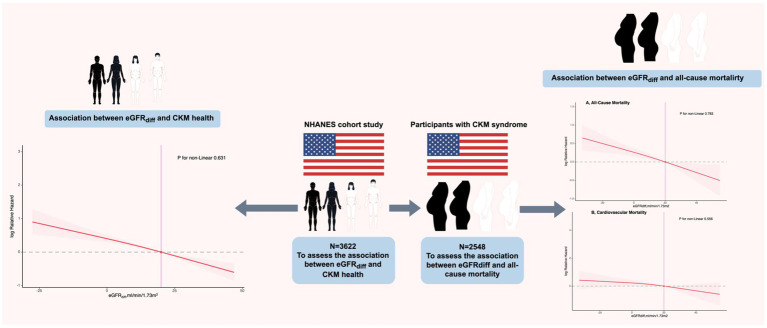

## 1 Introduction

Cardiorenal syndrome refers to the two-way relationship between heart and kidney dysfunction (1). Cardiometabolic disease is characterized by an increased risk of cardiovascular disease (CVD) due to issues with adipose tissue and metabolism (2). Recently, there has been growing awareness of the interactions between metabolic abnormalities, CVD, and kidney disease, and how they contribute to their development and progression (3). Metabolic abnormalities play a crucial role in the interaction between cardiovascular and kidney diseases, with kidney dysfunction acting as a significant mediator (4, 5). Poor cardiovascular–kidney–metabolic (CKM) health is linked to increased morbidity, multi-organ disease, and early death (4). To improve CKM health management, the American Heart Association (AHA) defined CKM stages in 2023 (4). Research based on this definition found that the prevalence of subclinical CKM syndrome was 80.94, 85.95, and 72.03% in the age groups 20–44 years, 45–64 years, and 65 years and older, respectively (6). The widespread prevalence of poor CKM health has serious public health implications. However, many treatments can improve CKM health (4). Therefore, we need to enhance screening for CKM health risk factors to support better staging and enable risk stratification for patients with CKM syndrome, thereby strengthening targeted preventive measures.

Estimated glomerular filtration rate (eGFR) based on serum creatinine (eGFRcreatinine) and cystatin C (eGFRcystatin) is commonly used to assess kidney function. In the majority of cases, eGFRcreatinine and eGFRcystatin are similar; however, there are notable differences in certain populations. In 2015, Grubb et al. (7) discovered that the eGFRcystatin/eGFRcreatinine ratio was significantly less than 1, with an elevated ratio of serum creatinine to cystatin C and other 11–29 kDa proteins. According to the pore model of glomerular filtration, this phenomenon is attributed to the fact that cystatin C (13,343 Da) and other 11–29 kDa proteins are considerably larger than creatinine (113 Da) (8). When renal filtration function declines, the filtration of larger molecules such as cystatin C and other proteins decreases before that of creatinine. Therefore, the difference between eGFRcystatin and eGFRcreatinine (eGFRcystatin—eGFRcreatinine, eGFRdiff) is considered an independent indicator of kidney function (9, 10). A study based on the Chronic Renal Insufficiency Cohort (CRIC) found that a reduction in eGFRdiff was associated with an increased risk of hospitalization for heart failure (HF) (11). Another study, using the eGFRcystatin/eGFRcreatinine ratio to measure the difference between eGFRcystatin and eGFRcreatinine, revealed that a reduced ratio was associated with a higher risk of 30-day re-hospitalization and mortality in heart failure (HF) patients, along with a decrease in their life quality (12). A recent study based on the UK Biobank found that a reduction in eGFRdiff was associated with an increased risk of atrial fibrillation (13). Furthermore, a decrease in the ratio of eGFRcystatin and eGFRcreatinine was not only related to an increase in atherosclerosis-related protein levels but also associated with the occurrence of atherosclerotic cardiovascular events (14, 15).

The objectives of this study were to examine the association between eGFRdiff and AHA-defined CKM health and to investigate the relationship between eGFRdiff and mortality in patients with CKM syndrome.

## 2 Materials and methods

### 2.1 Population

As cystatin C was measured exclusively during the 1999–2004 waves of the National Health and Nutrition Examination Survey (NHANES), our study analyzed data from 31,126 participants within this period. Since the Predicting Risk of cardiovascular diseases EVENTs (PREVENT) base model, which is used to define CKM, is applicable to U.S. individuals aged 30–79 years (16), our study only included subjects in this age group and excluded those with a classification of “Other Race—Including Multi-Racial.” In addition, we excluded individuals with missing variables required to define CKM (Supplementary Tables S1, S2), those without serum cystatin C and serum creatinine data, and pregnant women. The specific inclusion and exclusion criteria are presented in Supplementary Figure S1. Finally, 3,622 participants were included in our study. The NHANES program was approved by the National Center for Health Statistics (NCHS) Ethics Review Board, and all participants provided written informed consent (17). Since NHANES data are de-identified and anonymized during analysis, secondary analyses did not require additional ethical approval or informed consent.

### 2.2 Estimated glomerular filtration rate

Cystatin C levels were measured using a cystatin C immunoassay on an automated multi-channel analyzer (Siemens Dimension Vista 1,500, Siemens Healthcare Diagnostics) (18). For creatinine levels, an improved version of the Jaffé reaction, modified by Popper et al. and Seeling and Wuest, was used. Values below 0.6 mg/dL were considered insignificant and treated as missing data (19). Notably, there were discrepancies between the creatinine levels measured during the NHANES 1999–2000 and the gold standard reference method (i.e., the Roche coupled enzymatic assay performed on a Roche P Module instrument). Therefore, following the NHANES recommendations, Deming regression was applied to adjust the creatinine results for the NHANES 1999–2000 study (19).

eGFRcystatin was calculated using the CKD-EPI cystatin C (2012) equation, while eGFRcreatinine was calculated using the CKD-EPI creatinine (2009) equation (20, 21). The best eGFR (eGFRbest) is the average of eGFRcreatinine and eGFRcystatin (22). eGFRdiff is the difference between eGFRcystatin and eGFRcreatinine. eGFRdiff was analyzed as a continuous variable (per 10 mL/min/1.73 m2). Furthermore, participants were categorized into three groups according to their eGFRdiff: negative (<−15 mL/min/1.73 m2), midrange (−15 to 15 mL/min/ 1.73 m2), and positive (≥15 mL/min/1.73 m2) (13).

### 2.3 Cardiovascular–kidney–metabolic health

CKM Stage 0 comprised individuals who were not overweight (body mass index (BMI) <25 kg/m^2^) and had no metabolic risk factors (including hypertriglyceridemia [<135 mg/dL], hypertension, prediabetes, diabetes, and metabolic syndrome) or chronic kidney disease (CKD). CKM Stage 1 included individuals with a BMI ≥25 kg/m^2^, a waist circumference ≥ 88/102 cm in women/men, and/or prediabetes, without other metabolic risk factors or CKD. CKM Stage 2 included individuals with metabolic risk factors (including hypertriglyceridemia [≥135 mg/dL], hypertension, diabetes, and metabolic syndrome) or CKD. CKM Stage 3 included individuals with CKM and a 10-year cardiovascular disease (CVD) risk ≥20%, as predicted by the PREVENT base model (where the values for age and other risk factors outside the validated PREVENT ranges were imputed as the upper and lower limits of these ranges) (16). CKM Stage 4 included individuals with clinical CVD (coronary heart disease, congestive heart failure, and stroke) (4, 6). Hypertension was defined as a history of the disease or elevated blood pressure measurements. Diabetes and prediabetes were diagnosed based on disease history, measured blood glucose levels, and glycohemoglobin levels. Metabolic syndrome (MetS) was identified based on the harmonized criteria set by the International Diabetes Federation. CKD was defined using eGFRbest and disease history. A more detailed definition of the variables used to define the CKM stages is presented in Supplementary Tables S1, S2. We defined CKM syndrome as CKM stage ≥2.

### 2.4 Covariables

Data on age, gender, ethnicity, income level, and education level were collected using standardized demographic questionnaires (23). Data on smoking status were gathered through a smoking questionnaire, and physical activity was assessed through an activity questionnaire (23). The Healthy Eating Index (HEI) was calculated according to the HEI-2015 (24). The urine albumin-creatinine ratio (uACR, mg/g) is the ratio of urine albumin to creatinine. A more detailed definition of covariables is presented in Supplementary Table S3.

Data on all-cause and cardiovascular mortality status were obtained from the National Death Index (NDI). Cardiovascular mortality was defined using the International Classification of Diseases-10 (ICD-10) codes I00-I09, I11, I13, I20-I51, and I60-I69. Time was measured in months from the physical examination to either the date of death or the end of the follow-up period (31 December 2019).

### 2.5 Statistical analysis

Before analysis, we utilized the random forest imputation method to estimate the missing values, as the urine albumin-creatinine ratio had the highest proportion of missing values (13.94%, Supplementary Figure S2). According to the “highest proportion of missing values” principle, we performed complex sample analysis using fasting sample weights (25). Continuous variables were presented as weighted means and standard deviations (SDs), while categorical variables were expressed as weighted frequencies and proportions. The baseline characteristics were compared using eGFRdiff. The weighted Wilcoxon rank-sum test and the chi-squared test with Rao–Scott second-order corrections were used to compare group differences for continuous and categorical variables, respectively.

We first estimated the age-adjusted prevalence of each CKM stage by standardizing to the 2000 U.S. population census, which includes three age groups (30–39, 40–59, and 60–79 years) (26, 27). Then, we used ordinal logistic regression to explore the relationship between eGFRdiff and CKM stages. Model 1 was unadjusted for covariables. Model 2 included adjustments for age, gender, ethnicity, income level, educational level, smoking status, average physical activity level, HEI, and uACR. Model 3 was further adjusted for eGFRbest. To explore potential non-linear associations, we used restricted cubic splines (RCSs) in Model 3. The reference value was set at 20 mL/min/1.73m^2^, with the 10th, 50th, and 90th percentiles of eGFRdiff serving as the three knots for the spline. Subgroup analyses were further performed based on age (cutoff value of 65 years), gender, ethnicity, uACR, smoking status, and physical activity.

We further used the Cox regression model to explore the association between eGFRdiff and both all-cause mortality and cardiovascular mortality in the participants with and without CKM syndrome. Model 1 was unadjusted for covariables. Model 2 included adjustments for age, gender, ethnicity, income level, educational level, smoking status, average physical activity level, HEI, and uACR. Model 3 was further adjusted for eGFRbest. To explore potential non-linear associations, we used RCSs in Model 3. The reference value was set at 20 mL/min/1.73m^2^, with the 10th, 50th, and 90th percentiles of eGFRdiff serving as the three knots for the spline. Since the criteria for defining CKM stages include hypertension, blood glucose status, systolic blood pressure, triglycerides, and cholesterol, we excluded these variables from our models (28, 29).

Furthermore, we employed complete case analysis to avoid imputation bias for the missing data (*n* = 778). A two-sided *p*-value of <0.05 was considered statistically significant. Data analyses were performed using R 4.3.2.

## 3 Results

### 3.1 Baseline characteristics

Table 1 presents the baseline characteristics of the 3,622 participants in our study. The weighted mean age was 49.62 years (SD, 12.92 years); 50.74% of the participants were women, and 78.75% were non-Hispanic white. The unweighted proportions of the participants in CKM Stages 0–4 were as follows: 13.20% for Stage 0, 16.46% for Stage 1, 52.62% for Stage 2, 7.62% for Stage 3, and 10.10% for Stage 3.

**Table 1:** Baseline characteristics.

Characteristic	Sampled individual^1^	Population estimated^2^	Negative (<−15)^2^	Midrange (−15 to 15)^2^	Positive (≥15)^2^	*P*-value^3^
No. of participants	3,622	127,594,016	4,296,279	63,510,095	59,787,642	
Age in years	53.00 (41,65)	49.62 (12.92)	52.33 (12.63)	50.98 (13.62)	47.98 (11.95)	<0.001
Gender						<0.001
Women	1,775 (49.01%)	64,743,785 (50.74%)	2,254,559 (52.48%)	36,078,630 (56.81%)	26,410,597 (44.17%)	
Men	1,847 (50.99%)	62,850,231 (49.26%)	2,041,721 (47.52%)	27,431,465 (43.19%)	33,377,045 (55.83%)	
Ethnicity						0.034
Other Hispanic	153 (4.22%)	6,381,450 (5.00%)	170,796 (3.98%)	3,445,727 (5.43%)	2,764,926 (4.62%)	
Non-Hispanic white	1,947 (53.75%)	100,480,640 (78.75%)	3,153,835 (73.41%)	48,638,085 (76.58%)	48,688,720 (81.44%)	
Non-Hispanic Black	640 (17.67%)	12,694,862 (9.95%)	803,405 (18.70%)	6,819,122 (10.74%)	5,072,334 (8.48%)	
Mexican American	882 (24.35%)	8,037,064 (6.30%)	168,242 (3.92%)	4,607,160 (7.25%)	3,261,662 (5.46%)	
Income level						<0.001
Low	1,354 (37.38%)	61,653,605 (48.32%)	1,355,299 (31.55%)	25,708,816 (40.48%)	34,589,489 (57.85%)	
Median	1,383 (38.18%)	44,906,766 (35.20%)	1,649,976 (38.40%)	24,548,239 (38.65%)	18,708,552 (31.29%)	
High	885 (24.43%)	21,033,645 (16.48%)	1,291,004 (30.05%)	13,253,040 (20.87%)	6,489,601 (10.85%)	
Educational level						<0.001
Below high school	1,169 (32.27%)	24,616,150 (19.29%)	1,136,833 (26.46%)	15,392,665 (24.24%)	8,086,652 (13.53%)	
High school or higher	2,453 (67.73%)	102,977,866 (80.71%)	3,159,447 (73.54%)	48,117,430 (75.76%)	51,700,990 (86.47%)	
Smoking status						<0.001
Never	1,702 (46.99%)	59,048,209 (46.28%)	1,380,890 (32.14%)	25,580,040 (40.28%)	32,087,278 (53.67%)	
Former	1,098 (30.31%)	37,864,415 (29.68%)	1,120,630 (26.08%)	17,912,725 (28.20%)	18,831,060 (31.50%)	
Current	822 (22.69%)	30,681,393 (24.05%)	1,794,759 (41.77%)	20,017,330 (31.52%)	8,869,304 (14.83%)	
Healthy eating index	50.68 (12.82)	50.16 (12.83)	48.25 (11.24)	49.54 (12.89)	50.97 (12.83)	<0.001
Average level of physical activity						0.060
Mainly sitting	851 (23.50%)	31,599,427 (24.77%)	1,630,622 (37.95%)	15,556,303 (24.49%)	14,412,502 (24.11%)	
Walking a lot	2,009 (55.47%)	66,765,431 (52.33%)	2,100,908 (48.90%)	33,919,770 (53.41%)	30,744,752 (51.42%)	
Carrying light loads	529 (14.61%)	20,445,567 (16.02%)	398,314 (9.27%)	9,569,697 (15.07%)	10,477,555 (17.52%)	
Carrying heavy loads	233 (6.43%)	8,783,592 (6.88%)	166,435 (3.87%)	4,464,324 (7.03%)	4,152,833 (6.95%)	
Body mass index, kg.m2	28.80 (6.00)	28.50 (6.12)	31.28 (8.23)	29.04 (6.73)	27.73 (5.06)	<0.001
Waist circumference, cm	98.80 (14.61)	97.98 (15.28)	105.50 (19.22)	99.17 (16.09)	96.18 (13.73)	<0.001
Systolic blood pressure	127.32 (20.21)	123.65 (18.20)	130.01 (19.15)	125.04 (18.98)	121.71 (17.00)	<0.001
Diastolic blood pressure	72.82 (12.88)	73.05 (12.07)	73.33 (14.96)	72.41 (12.76)	73.72 (11.02)	0.12
Hypertension						<0.001
No	1,973 (54.47%)	77,397,206 (60.66%)	2,178,268 (50.70%)	36,260,339 (57.09%)	38,958,598 (65.16%)	
Yes	1,649 (45.53%)	50,196,810 (39.34%)	2,118,011 (49.30%)	27,249,756 (42.91%)	20,829,044 (34.84%)	
Blood glucose status						<0.001
Diabetes	489 (13.50%)	13,718,955 (10.75%)	1,309,216 (30.48%)	7,418,968 (11.68%)	4,990,772 (8.34%)	
Prediabetes	325 (8.97%)	9,188,042 (7.20%)	312,105 (7.26%)	5,315,238 (8.37%)	3,560,699 (5.96%)	
Normal	2,808 (77.53%)	104,687,019 (82.05%)	2,674,958 (62.26%)	50,775,889 (79.95%)	51,236,171 (85.70%)	
Cardiovascular disease						<0.001
No	3,256 (89.90%)	117,361,581 (91.98%)	3,624,243 (84.36%)	57,136,056 (89.96%)	56,601,282 (94.67%)	
Yes	366 (10.10%)	10,232,435 (8.02%)	672,036 (15.64%)	6,374,039 (10.04%)	3,186,360 (5.33%)	
Chronic kidney disease						<0.001
No	3,405 (94.01%)	121,627,069 (95.32%)	3,964,746 (92.28%)	58,947,947 (92.82%)	58,714,376 (98.20%)	
Yes	217 (5.99%)	5,966,947 (4.68%)	331,533 (7.72%)	4,562,148 (7.18%)	1,073,266 (1.80%)	
Cholesterol, mg/dl	201 (178,227)	204.15 (40.43)	199.79 (42.15)	204.76 (40.32)	203.82 (40.42)	0.6
High-density lipoprotein, mg/dl	51.77 (15.55)	52.04 (15.59)	47.83 (17.35)	50.99 (15.44)	53.45 (15.48)	<0.001
Triglyceride, mg/dl	115 (79,170)	141.40 (141.05)	157.67 (119.65)	154.38 (171.42)	126.44 (99.05)	<0.001
uACR, mg/g	5.67 (3.93,9.17)	6.80 (4.60)	7.74 (5.11)	7.45 (4.86)	6.06 (4.14)	<0.001

1 Unweighted mean (SD) or unweighted median (IQR); unweighted n(%).

2 Weighted mean (SD); weighted n (%).

3 Wilcoxon rank-sum test for the complex survey samples; the chi-squared test with Rao–Scott second-order corrections.

eGFR diff, the difference between cystatin C–based estimated glomerular filtration rate and creatinine-based estimated glomerular filtration rate; uACR, urine albumin-creatinine ratio; CKM, cardiovascular–kidney–metabolic syndrome.

Compared to the participants with the midrange eGFRdiff, those with the negative eGFRdiff were more likely to be current smokers and had higher levels of body mass index (BMI), waist circumference, systolic blood pressure (SBP), and triglycerides, in addition to higher rates of hypertension, diabetes, cardiovascular disease (CVD) and chronic kidney disease (CKD) stage (all *p* < 0.001). Conversely, participants with positive eGFRdiff showed better health indicators, including BMI, waist circumference, SBP, triglycerides, high-density lipoprotein, hypertension, diabetes, CVD, and CKD stage (all *p* < 0.001).

### 3.2 Association between eGFRdiff and CKM stages

The prevalence of higher CKM stages was significantly higher in participants with negative eGFRdiff and lower in those with positive eGFRdiff (Supplementary Table S4). The age-adjusted prevalence rates for CKM Stage 3 were 10.06% [95% confidence interval (CI), 3.31 to 16.81] in the negative eGFRdiff group and 3.23% [95% CI, 2.22 to 4.24] in the positive eGFRdiff group. For CKM Stage 4, the rates were 14.9% [95% CI, 6.35 to 23.44] in the negative eGFRdiff group and 6.43% [95% CI, 5.08 to 7.78] in the positive eGFRdiff group.

**Figure 1:**
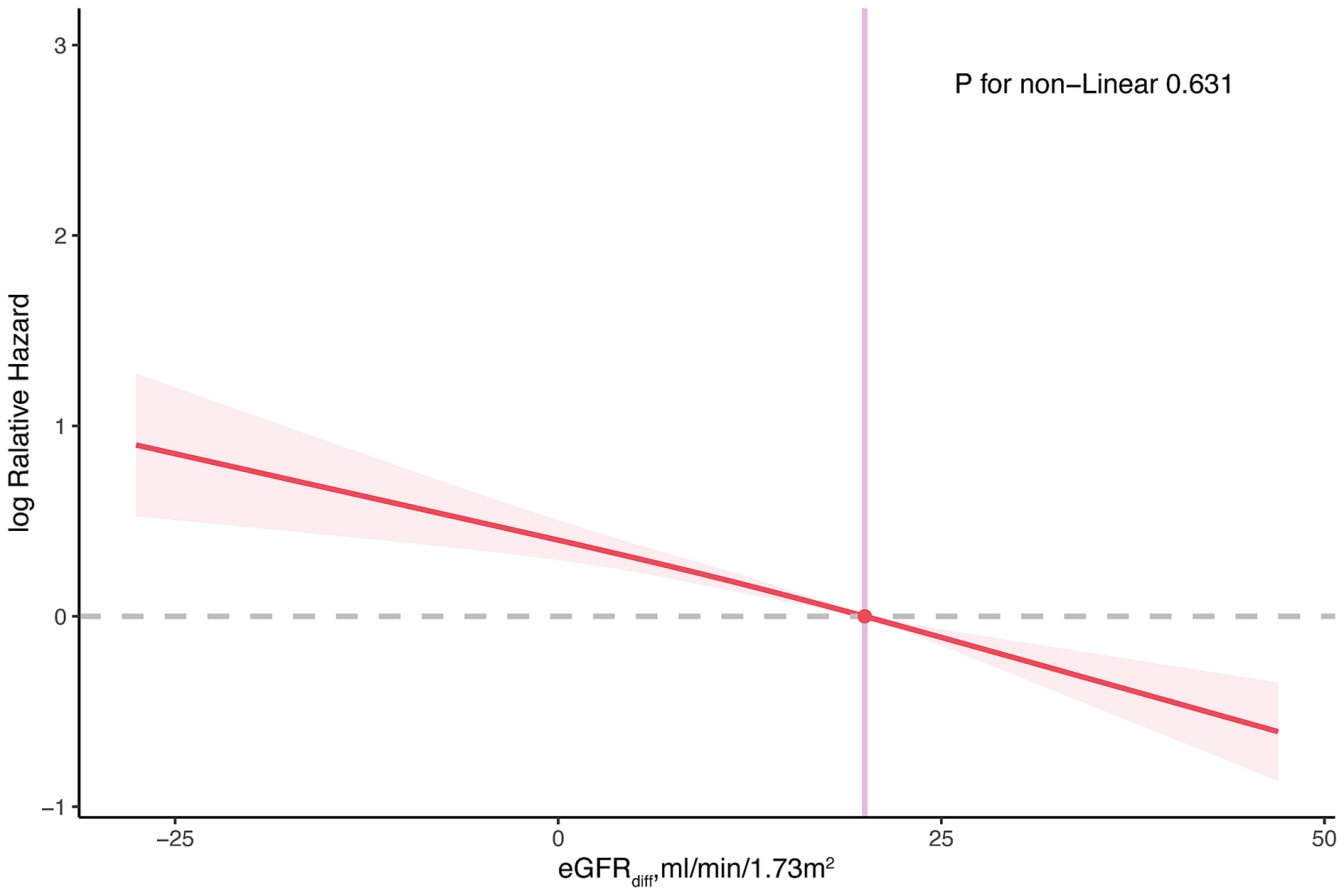
Restricted cubic spline plot for the association between eGFRdiff and cardiovascular–kidney–metabolic syndrome. The restricted cubic spline model was adjusted for age, gender, ethnicity, income level, educational level, smoking status, average level of physical activity, Healthy Eating Index, urine albumin-creatinine ratio, and estimated glomerular filtration rate. eGFR diff, the difference between cystatin C–based estimated glomerular filtration rate and creatinine-based estimated glomerular filtration rate.

When eGFRdiff was treated as a continuous variable, the common odds ratio (cOR) per 10 mL/min/1.73m^2^ increase in eGFRdiff in the fully adjusted model was 0.86 [95% confidence interval (CI), 0.81 to 0.91] (Table 2). Compared to the midrange eGFRdiff, the cOR values for the negative and positive eGFRdiff were 1.88 [95% CI, 1.23 to 2.88] and 0.69 [95% CI, 0.58 to 0.83], respectively (Table 2). RCS analysis suggested no significant non-linear association between eGFRdiff and CKM stages (Figure 1).

**Table 2:** Common odd ratios (cORs) for the association between eGFR difference and cardiovascular–kidney–metabolic syndrome.

eGFR_diff_	Model 1	*P*-Value	Model 2	*P*-Value	Model 3	*P*-Value
Continuous value						
Per 10 mL/min/1.73m^2^	0.81 [0.76,0.86]	<0.001	0.85 [0.82,0.89]	<0.001	0.86 [0.81,0.91]	<0.001
Categorical value						
Negative (≤ − 15 mL/min/1.73 m^2^)	2.42 [1.62,3.6]	<0.001	2.23 [1.47,3.39]	<0.001	1.88 [1.23,2.88]	0.004
Midrange (−15 to 15 mL/min/1.73 m^2^)	Ref	-	Ref	-	Ref	-
Positive (>15 mL/min/1.73 m^2^)	0.59 [0.49,0.71]	<0.001	0.72 [0.6,0.86]	<0.001	0.69 [0.58,0.83]	<0.001

eGFR diff, the difference between cystatin C–based estimated glomerular filtration rate and creatinine-based estimated glomerular filtration rate. Model 1 was unadjusted; Model 2 was adjusted for age, gender, ethnicity, income level, educational level, smoking status, average level of physical activity, Healthy Eating Index, and urine albumin-creatinine ratio; and Model 3 was adjusted for Model 2 + the estimated glomerular filtration rate.

The association between eGFRdiff and CKM stages can be found in the majority of the subgroups (Supplementary Figure S3). The cOR values for the participants aged <65 years, female participants, male participants, non-Hispanic white participants, and participants who had never smoked were 0.82 [95%CI, 0.75 to 0.89], 0.83[95%CI, 0.75 to 0.92], 0.89[95%CI, 0.83 to 0.95], 0.86[95%CI, 0.8 to 0.92], and 0.78[95%CI, 0.72 to 0.84], respectively. Although not statistically significant (*p* = 0.746), the cOR for the participants aged 65 years and older was 1.02 [95% CI, 0.92 to 1.12].

### 3.3 Association between eGFRdiff and both all-cause and cardiovascular mortality

**Figure 2:**
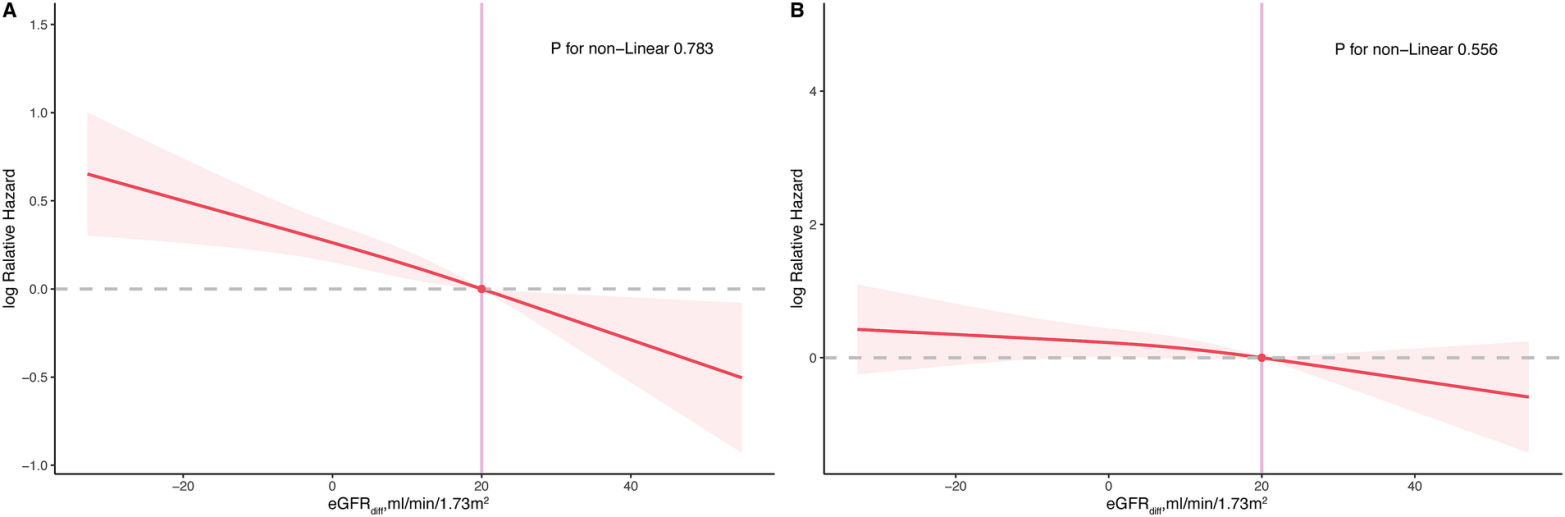
Restricted cubic spline plot for the association between eGFRdiff and both all-cause and cardiovascular mortality in participants with cardiovascular–kidney–metabolic (CKM) syndrome. **(A)**, all-cause mortality; **(B)**, cardiovascular mortality.

During the median follow-up period of 201 months, 853 participants (853/2548 participants with CKM syndrome, 33.48%) experienced all-cause mortality, and 265 participants (265/2548 participants with CKM syndrome, 10.4%) experienced cardiovascular mortality. In the fully adjusted model, for participants with CKM syndrome, the hazard ratios (HRs) per 10 mL/min/1.73m^2^ increase in eGFRdiff were 0.88 [95% CI, 0.83 to 0.93] for all-cause mortality and 0.90 [95%CI, 0.81 to 1.00] for cardiovascular mortality, with the latter being marginally significant (*p* = 0.052) (Table 3). Compared to participants with midrange eGFRdiff, those with negative eGFRdiff had a 48% increased risk of all-cause mortality, while those with positive eGFRdiff had a 30% reduced risk of all-cause mortality. There was no significant difference in cardiovascular mortality risk between participants with negative or positive eGFRdiff and those with midrange eGFRdiff (Table 3). The RCS analysis indicated no significant non-linear association between eGFRdiff and both all-cause mortality and cardiovascular mortality in participants with and without CKM syndrome (Figure 2).

**Table 3:** Impact of eGFR_diff_ on both all-cause and cardiovascular mortality in participants with cardiovascular–kidney–metabolic syndrome.

	All-cause mortality			Cardiovascular-cause mortality		
eGFRdiff	Model 1	Model 2	Model 3	Model 1	Model 2	Model 3
Continuous value						
Per 10 mL/min/1.73m^2^	0.80 [0.75,0.85]	0.86 [0.81,0.90]	0.88 [0.83,0.93]	0.80 [0.73, 0.89]	0.86 [0.77,0.96]	0.90 [0.81,1.00]
*P*-Value	<0.001	<0.001	<0.001	<0.001	0.006	0.052
Categorical value						
Negative (≤ − 15 mL/min/1.73 m^2^)	1.56 [1.02,2.38]	1.57 [1.08,2.27]	1.48 [1.03,2.14]	1.51 [0.70, 3.25]	1.61 [0.79,3.28]	1.50 [0.74,3.01]
*P*-Value	0.042	0.017	0.033	0.295	0.189	0.259
Midrange (−15 to 15 mL/min/1.73 m^2^)	Ref	Ref	Ref	Ref	Ref	Ref
*P*-Value	-	-	-	-	-	-
Positive (>15 mL/min/1.73 m^2^)	0.52 [0.44,0.61]	0.66 [0.55,0.80]	0.70 [0.58,0.85]	0.54 [0.38, 0.75]	0.71 [0.50,1.01]	0.78 [0.55,1.10]
*P*-Value	<0.001	<0.001	<0.001	<0.001	0.054	0.155

eGFR diff, the difference between cystatin C–based estimated glomerular filtration rate and creatinine-based estimated glomerular filtration rate. Model 1 was unadjusted; Model 2 was adjusted for age, gender, ethnicity, income level, educational level, smoking status, average level of physical activity, Healthy Eating Index, and urine albumin-creatinine ratio; and Model 3 was adjusted for Model 2 + the estimated glomerular filtration rate.

### 3.4 Complete case analysis

After further excluding 778 participants with missing covariate data (Supplementary Figure S2), a total of 2,844 participants were included in the complete case analysis. The cOR per 10 mL/min/1.73m^2^ increase in eGFRdiff in the fully adjusted model was 0.84 [95% CI, 0.79 to 0.89] (Supplementary Table S5). Compared to participants with midrange eGFRdiff, those with negative eGFRdiff had a cOR of 1.97 [95% CI, 1.1 to 3.52] and those with positive eGFRdiff had a cOR of 0.66 [95% CI, 0.54 to 0.79] (Supplementary Table S5). No significant non-linear association was detected (Supplementary Figure S4).

During a median follow-up of 204 months, 538 participants included in the complete-case analysis experienced all-cause mortality and 159 participants experienced cardiovascular mortality. In the fully adjusted model, the hazard ratios (HRs) per 10 mL/min/1.73m^2^ increase in eGFRdiff were 0.87 [95% CI, 0.82 to 0.93] and 0.97 [95% CI, 0.89 to 1.07] for all-cause mortality and cardiovascular mortality (Supplementary Table S6), respectively. No significant non-linear association was detected (Supplementary Figure S5).

## 4 Discussion

This study used a large prospective cohort from the NHANES to investigate the association between eGFRdiff and CKM health, along with the relationship between eGFRdiff and mortality in CKM syndrome patients. An increase in eGFRdiff was associated with an improvement in CKM health. The participants with negative eGFRdiff tended to have poorer CKM health, whereas those with positive eGFRdiff tended to have better CKM health. In addition, an increase in eGFRdiff was related to a lower risk of all-cause mortality in CKM syndrome patients, while the risk of cardiovascular mortality in these patients was marginally significant. These associations were independent of the uACR and eGFRbest-assessed kidney function.

According to the KDIGO guidelines, the primary criteria for diagnosing CKD include a measured or estimated decline in the GFR and/or significant proteinuria (30). However, multiple studies have shown that in patients without kidney disease according to the KDIGO standards, a significant discrepancy between eGFRcystatin and eGFRcreatinine is associated with a substantial increase in the risk of comorbidities and mortality (12, 14, 31–37). This suggests that the discrepancy between eGFRcystatin and eGFRcreatinine, independent of eGFRcreatinine, eGFRcystatin, and uACR, is a risk factor for kidney function impairment. Numerous studies have investigated the relationship between eGFRdiff and CKM health-related factors and adverse cardiovascular events. A multicenter prospective cohort study explored the association between eGFRdiff and end-stage kidney disease (ESKD) and mortality in patients with mild-to-moderate CKD. It was found that patients with negative eGFRdiff had an 86% increased risk of mortality compared to those with midrange eGFRdiff, while those with positive eGFRdiff had a 27% lower risk of ESKD and a 32% lower risk of mortality (38). A Korean study examined the correlation between eGFRdiff and major adverse cardiovascular events (MACE) and coronary artery calcification (CAC) in CKD patients. It was found that patients in the highest tertile of baseline eGFRdiff had a 2.12 times higher risk of MACE compared to those in the lowest tertile, and they also had higher baseline CAC and more significant CAC progression (15). Based on the CRIC study, Chen et al. used Fine–Gray proportional sub-distribution hazards regression to investigate the association between eGFRdiff and HF hospitalization. They found that for every 15 mL/min/1.73m^2^ decrease in eGFRdiff, the risk of HF hospitalization increased by 20%, independent of eGFRcystatin and eGFRcreatinine (11). In addition, a study based on a Swedish HF inpatient population found that shrunken pore syndrome (eGFRcystatin/eGFRcreatinine <0.6) was associated with decreased quality of life, increased risk of all-cause mortality, and higher risk of 30-day rehospitalization (12). Furthermore, a study of diabetic patients found that each SD decrease in eGFRdiff was associated with a 28% increased risk of overall diabetic microvascular complications, a 14% higher risk of diabetic retinopathy, and a 29% higher risk of diabetic kidney disease (39). One possible mechanism is that a significant increase in eGFRdiff indicates reduced kidney clearance of medium molecular weight substances, leading to the accumulation of atherosclerotic proteins and other inflammatory factors (7, 14, 32). In addition, eGFRcystatin and eGFRcreatinine are influenced by different non-renal factors. Age, decreased physical activity, and reduced muscle mass may lead to an overestimation of eGFRcreatinine, while eGFRcystatin is affected by thyroid function, inflammation levels, and obesity (40).

Our study offers two major clinical implications. First, eGFRdiff is significantly associated with CKM health and correlates with mortality risk in CKM syndrome patients. Therefore, assessing CKM health using only eGFRcystatin or eGFRcreatinine and proteinuria levels is insufficient. Second, eGFRcreatinine and eGFRcystatin should be reported separately. Both our study and previous research indicate that eGFRdiff provides important prognostic information. In addition, studies have suggested that calculating eGFRcreatinine and eGFRcystatin separately and then averaging them to obtain the final eGFR is the best method for estimating kidney function (22).

Furthermore, there is currently no clear definition of which CKM stage should be used as a threshold to classify patients as having or not having CKM syndrome. Unlike our study, which used CKM Stage 2 as the threshold, previous studies used CKM Stage 3 (41). Their research explored the relationship between CKM syndrome and adverse events following major non-cardiac surgery. This classification was made for clinical reasons, as Stage 3 is the first stage to include both subclinical cardiovascular disease and CKD, making it a potentially critical point for adjusting perioperative management. However, our study population consisted of community residents, mainly focusing on primary and secondary prevention, making Stage 3 too stringent a threshold. Metabolic abnormalities in Stage 1 include being overweight (BMI ≥25 kg/m^2^), having an increased waist circumference (≥88/102 cm in women/men), or having prediabetes but no other metabolic risk factors or CKD. Individuals in Stage 1 are at some risk but do not yet exhibit multiple metabolic disorders or organ damage, which is insufficient to define the condition as a syndrome. Individuals in Stage 2 have significant metabolic risk factors or CKD that substantially increase the risk of cardiovascular and other diseases. Active intervention at this stage can effectively prevent disease progression and offers a high clinical intervention value. Therefore, for community residents focused on primary and secondary prevention, using Stage 2 as a threshold is more appropriate. In addition, the AHA’s Presidential Advisory defines Stage 3 of the CKM syndrome as “subclinical CVD among individuals with excess/dysfunctional adiposity, metabolic risk factors, or CKD,” further validating our use of Stage 2 as the threshold for the presence of the CKM syndrome (4).

This study has several strengths, such as a long follow-up period (median of 205 months), which allowed for the assessment of mortality over a significant period of time. To the best of our knowledge, this is the first study to examine the association between eGFRdiff and CKM health and mortality risk in CKM syndrome patients. However, our study also has several limitations. First, due to its retrospective nature, there were potential confounding factors. For instance, although some studies have suggested that using the average of eGFRcreatinine and eGFRcystatin provides a more accurate final eGFR (22), we cannot completely rule out the bias between eGFRbest and the measured values. Second, creatinine and cystatin C were only measured at baseline, limiting our ability to assess changes in eGFRdiff and their impact on CKM health and mortality in CKM syndrome patients. Third, we only examined the absolute difference between eGFRcystatin and eGFRcreatinine, rather than the relative difference, because, in our experience, clinicians are more accustomed to using the absolute difference. Fourth, due to the lack of data on subclinical atherosclerotic cardiovascular disease, subclinical heart failure, peripheral arterial disease, and atrial fibrillation, we relied on the PREVENT base model from previous literature (6), which may have led to an underestimation of Stage 3. Fifth, although eGFRdiff provides a more comprehensive assessment of CKD health and prognosis, cost constraints mean that eGFRcreatinine and proteinuria levels remain essential and practical indicators in solutions where measuring cystatin C is not feasible.

## 5 Conclusion

Our study found that eGFRdiff is associated with CKM health and serves as a risk stratification factor for long-term all-cause mortality in patients with CKM syndrome, independent of eGFR and uACR. These findings suggest that eGFRdiff should be considered when assessing CKM health, rather than relying solely on proteinuria, eGFRcystatin, and eGFRcreatinine.

## Data Availability

Publicly available datasets were analyzed in this study. This data can be found here: the National Health and Nutrition Examination Surveys (NHANES) at: https://www.cdc.gov/nchs/nhanes/index.htm.
