# Supplementary material for: Difference between estimated glomerular filtration rate based on cystatin C versus creatinine and cardiovascular–kidney–metabolic health
Source: Front Med (Lausanne). 2025 Jan 15;11:1477343. doi: 10.3389/fmed.2024.1477343 (PMC11774968; doi:10.3389/fmed.2024.1477343)
Supplement: Supplementary file 1 [file Supplementary_file_1.docx]

Supplementary Material

# Supplementary Figures and Tables

## Supplementary Tables

Table S1 Definition of the variables used to define the CKM stage

| Variables | Definition |
| --- | --- |
| Smoking status(1) | Current smoking: smoked moth than 100 cigarettes in life and smoke some days or every day  Former smoking: smoked more than 100 cigarettes in life and smoke not at all now  Never smoking: smoked less than 100 cigarettes in life |
| Hypertension | Being previously diagnosed with hypertension by a healthcare professional; (2) or  Having a blood pressure (BP) reading ≥140/90mmHg on at least three out of four measurements; or  Using anti-hypertensive medications at baseline. |
| Blood glucose status | Diabetes:  Being previously diagnosed with diabetes by a healthcare professional; (2) or  Having a glycohemoglobin level >6.5%, fasting glucose level ≥7.0 mmol/L random blood glucose level ≥11.1 mmol/L, two-hour oral glucose tolerance test (OGTT) blood glucose level ≥11.1 mmol/L; or  Using diabetes medications or insulin at baseline.  Pre-diabetes (including Impaired Fasting Glycaemia and Impaired Glucose Tolerance)   1. Impaired Fasting Glycaemia (IFG): 7.0 mmol/L> fasting glucose level ≥6.1 mmol/L 2. Impaired Glucose Tolerance (IGT): 11.1 mmol/L> two-hour OGTT blood glucose level ≥7.7 mmol/L |
| Metabolic Syndrome(3, 4) | Metabolic syndrome was deﬁned as meeting 3 or more of the harmonized International Diabetes Federation criteria:  (1) elevated waist circumference greater than 102 cm for men or greater than 88 cm for women;  (2) triglyceride level greater than or equal to 150 mg/dL;  (3) high-density lipoprotein C level less than 40 mg/dL for men or less than 50 mg/dL for women;  (4) systolic blood pressure greater than or equal to 130 mm Hg, diastolic blood pressure greater than or equal to 85 mm Hg, or antihypertensive treatment in a patient with a history of hypertension;  (5) fasting glucose level greater than or equal to 100 mg/dL or drug treatment of elevated glucose level |
| Chronic kidney disease | eGFR <60 ml/min/1.73m^2^;or  Being previously diagnosed with weak/failing kidney function by a healthcare professional (2) |

Table S2 Examples of the calculation of predicted 10-year CVD risk(5)

| Risk factors | Original input value | Predictor value after required transformation | Coefficient | Product of predictor value and coefficient |
| --- | --- | --- | --- | --- |
| Age, year | 50 | (50 – 55) /10 = -0.5 | 0.7939329 | -0.39697 |
| Total cholesterol, mg/dL* | 200 | used to calculate non-HDL cholesterol |  |  |
| non-HDL cholesterol | --- | (200 – 45) × 0.02586 – 3.5 = 0.5083 | 0.0305239 | 0.015515 |
| HDL cholesterol, mg/dL* | 45 | (45 × 0.02586 – 1.3) /0.3 = -0.45433 | -0.16069 | 0.073005 |
| Systolic BP, mmHg | 160 |  |  |  |
| Systolic BP<110, mmHg | --- | (min(160,110) – 110) /20 = 0 | -0.2394 | 0 |
| Systolic BP 110+, mmHg | --- | (max(160,110) – 130) /20 = 1.5 | 0.360078 | 0.540117 |
| Diabetes mellitus | 1 | 1 | 0.86676 | 0.86676 |
| Current smoking | 0 | 0 | 0.536074 | 0 |
| eGFR, ml/min/1.73m^2^ | 90 |  |  |  |
| eGFR <60, ml | --- | (min(90,60) – 60) /-15 = 0 | 0.604592 | 0 |
| eGFR 60+, ml | --- | (max(90,60) – 90) /-15 = 0 | 0.043377 | 0 |
| HTN meds | 1 | 1 | 0.315167 | 0.315167 |
| Statin | 0 | 0 | -0.14777 | 0 |
| Treated SBP 110+ | spline | 1.5 | -0.06636 | -0.09954 |
| Treated non-HDLC | spline | 0 | 0.119788 | 0 |
| Age × non-HDL cholesterol | interaction | -0.5 × 0.5083 = -0.25415 | -0.08197 | 0.020833 |
| Age × HDL cholesterol | interaction | -0.5 × -0.45433 = 0.227167 | 0.030677 | 0.006969 |
| Age × Systolic BP 110+ | interaction | -0.5 × 1.5 = -0.75 | -0.09463 | 0.070976 |
| Age × Diabetes mellitus | interaction | -0.5 × 1 = -0.5 | -0.27057 | 0.135285 |
| Age × Current smoking | interaction | -0.5 × 0 = 0 | -0.07872 | 0 |
| Age × eGFR <60 | interaction | -0.5 × 0 = 0 | -0.16378 | 0 |
| Constant |  |  | -3.30773 | -3.30773 |
| Sum |  |  |  | -1.75961 |
| Risk = exp(-1.75961) / (1 + exp(-1.75961)) = 0.146839 = 14.7% | | | | |

*The equations use cholesterol in mg/dL and apply unit conversion (1 mmol/L = 0.02586 mg/dL)

Table S3 Definition of covariables

| Covariables | Definition |
| --- | --- |
| Ethnicity(6) | Classified as Mexican American, Non-Hispanic Black, Non-Hispanic White, or other |
| Income levels(7) | Low income: poverty to income ratio (PIR) ≤ 1.3  Median income: 3.5≥PIR>1.3  High income: PIR>3.5 |
| Educational levels(6) | Educational levels were categorized as less than high school and equal to or above high school. |
| Smoking status(1) | Current smoking: smoked moth than 100 cigarettes in life and smoke some days or every day  Former smoking: smoked more than 100 cigarettes in life and smoke not at all now  Never smoking: smoked less than 100 cigarettes in life |
| Physical Activity(8) | According to average level of physical activity each day  Mainly sit (MET=1.4): Sit during the day and do not walk about very much  Walk a lot (MET=1.5): stand or walk about a lot during the day, but do not have to carry or lift things very often  Carry light loads (MET=1.6): lift light load or have to climb stairs or hills often  Carry heavy loads (MET=1.8): do heavy work or carry heavy loads |

MET, metabolic equivalent score

Table S4 Prevalence Rates of Cardiovascular-Kidney-Metabolic Syndrome Stages According to 3 categories of eGFR_diff_ levels

|  | eGFR_diff_, ml/min/1.73m2 | | |
| --- | --- | --- | --- |
| Cardiovascular-Kidney-Metabolic Syndrome Stages | Negative (<−15) | Midrange (−15 to 15) | Positive (≥15) |
| Weighted Prevalence Rates^*^ |  |  |  |
| Stage 0 | 190,189 (4.43%) | 9,67 0,040 (15.23%) | 12,001,836 (20.07%) |
| Stage 1 | 301,349 (7.01%) | 9,595,262 (15.11%) | 12,906,099 (21.59%) |
| Stage 2 | 2,630,882 (61.24%) | 34,202,275 (53.85%) | 30,134,604 (50.40%) |
| Stage 3 | 501,822 (11.68%) | 3,668,480 (5.78%) | 1,558,744 (2.61%) |
| Stage 4 | 672,036 (15.64%) | 6,374,039 (10.04%) | 3,186,360 (5.33%) |
| Age-Adjusted Prevalence Rates, % |  |  |  |
| Stage 0 | 4.82[-0.69,10.33] ^§^ | 14.58[12.2,16.96] | 17.65[14.96,20.35] |
| Stage 1 | 6.85[2.61,11.08] | 14.95[12.38,17.51] | 19.75[17.26,22.25] |
| Stage 2 | 63.37[51.11,75.64] | 55.5[51.66,59.33] | 52.94[49.47,56.4] |
| Stage 3 | 10.06[3.31,16.81] | 5.01[3.96,6.07] | 3.23[2.22,4.24] |
| Stage 4 | 14.9[6.35,23.44] | 9.96[8.18,11.74] | 6.43[5.08,7.78] |

*present as weighted n(%)

^§^ It's possible that the confidence interval estimate is incorrect due to insufficient sample size (6/3622 unweighted sample), and should be cautious in consideration.

Table S5 Common odd ratios (cOR) for the association between eGFR difference and cardiovascular-kidney-metabolic syndrome in complete-case analysis

| eGFR_diff_ | Model1 | P Value | Model2 | P Value | Model3 | P Value |
| --- | --- | --- | --- | --- | --- | --- |
| Continuous |  |  |  |  |  |  |
| Per 10 ml/min/1.73m^2^ | 0.8[0.75,0.85] | <0.001 | 0.84[0.8,0.88] | <0.001 | 0.84[0.79,0.89] | <0.001 |
| Categorical Value |  |  |  |  |  |  |
| Negative (≤15 mL/min/1.73 m^2^) | 2.33[1.46,3.72] | <0.001 | 2.25[1.3,3.91] | 0.004 | 1.97[1.1,3.52] | 0.023 |
| Midrange (-15 to 15 mL/min/1.73 m2 ) | Ref | - | Ref | - | Ref | - |
| Positive (>15 mL/min/1.73 m^2^) | 0.58[0.47,0.7] | <0.001 | 0.69[0.57,0.83] | <0.001 | 0.66[0.54,0.79] | <0.001 |

eGFR_diff_; the difference between cystatin C–based estimated glomerular ﬁltration rate and creatinine based estimated glomerular ﬁltration rate. Model 1 was unadjusted; Model 2 was adjusted for age, gender, ethnicity, income level, educational level, smoking status, average level of physical activity, healthy eating index, and urine albumin-to-creatinine ratio; Model 3 was adjusted for Model 2+estimated glomerular filtration rate.

Table S6 Risk of eGFR_diff_ on both all-cause and cardiovascular mortality in participants with and without cardiovascular-kidney-metabolic syndrome in complete-case analysis.

|  | All-Cause Mortality | | | Cardiovascular Mortality | | |
| --- | --- | --- | --- | --- | --- | --- |
| eGFRdiff | Model1 | Model2 | Model3 | Model1 | Model2 | Model3 |
| Continuous |  |  |  |  |  |  |
| Per 10 ml/min/1.73m^2^ | 0.78 [0.73, 0.84] | 0.86 [0.81, 0.91] | 0.87 [0.82, 0.93] | 0.86 [0.78, 0.96] | 0.95 [0.87, 1.04] | 0.97 [0.89, 1.07] |
| P Value | <0.001 | <0.001 | <0.001 | 0.005 | 0.234 | 0.587 |
| Categorical Value |  |  |  |  |  |  |
| Negative  (≤-15 mL/min/1.73 m^2^) | 1.31 [0.77, 2.24] | 1.30 [0.78, 2.18] | 1.26 [0.75, 2.13] | NA | NA | NA |
| P Value | 0.325 | 0.32 | 0.386 | NA | NA | NA |
| Midrange  (-15 to 15 mL/min/1.73 m^2^) | Ref | Ref | Ref | Ref | Ref | Ref |
| P Value | - | - | - | - | - | - |
| Positive  (>15 mL/min/1.73 m^2^) | 0.48 [0.39, 0.58] | 0.63 [0.51, 0.78] | 0.65 [0.52, 0.80] | NA | NA | NA |
| P Value | <0.001 | <0.001 | <0.001 | NA | NA | NA |

eGFR diff; the difference between cystatin C–based estimated glomerular ﬁltration rate and creatinine based estimated glomerular ﬁltration rate. Model 1 was unadjusted; Model 2 was adjusted for age, gender, ethnicity, income level, educational level, smoking status, average level of physical activity, healthy eating index, and urine albumin-to-creatinine ratio; Model 3 was adjusted for Model 2+estimated glomerular filtration rate.

## Supplementary Figures


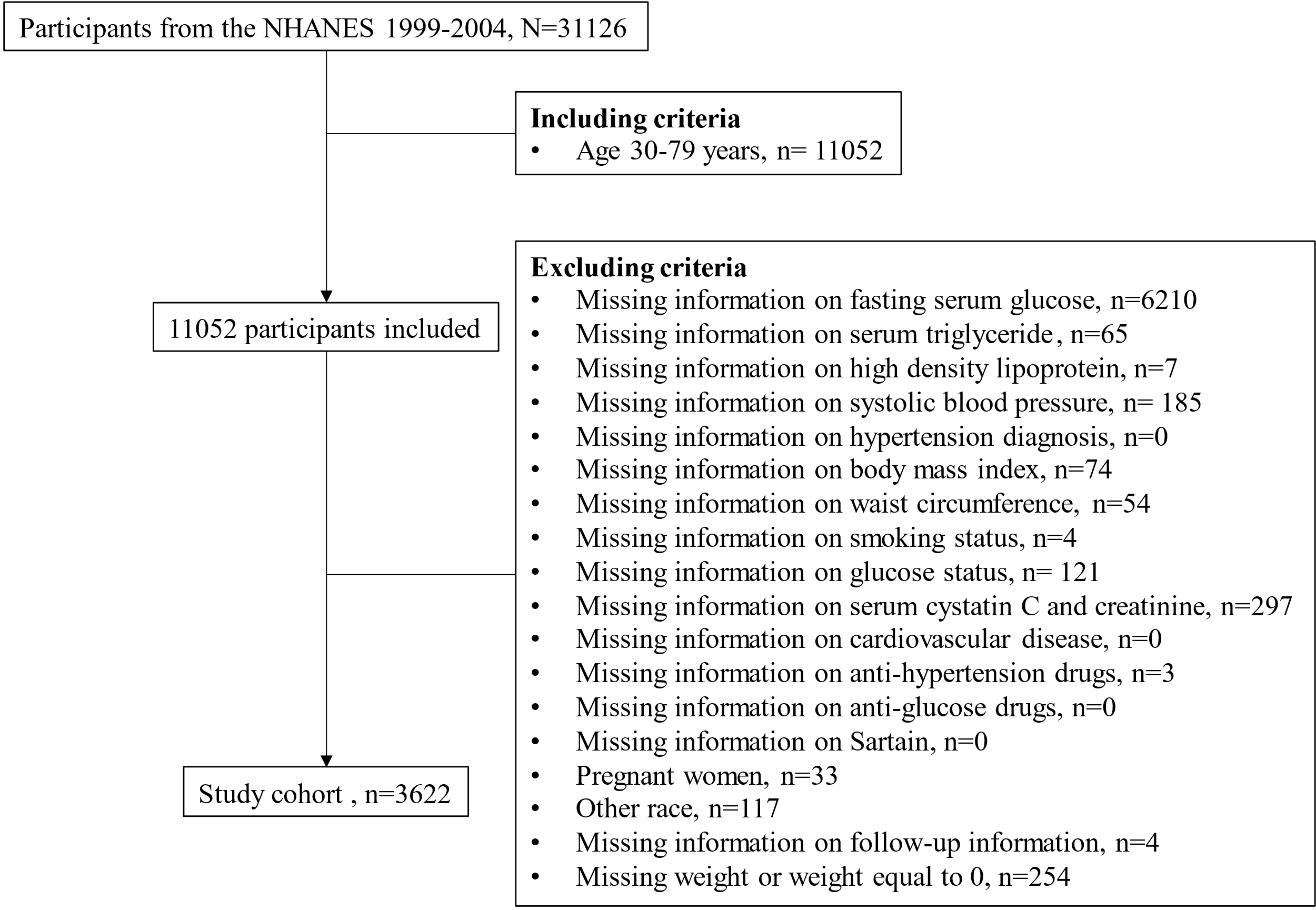


**Supplementary Figure S1.** Flowchart of participants selection


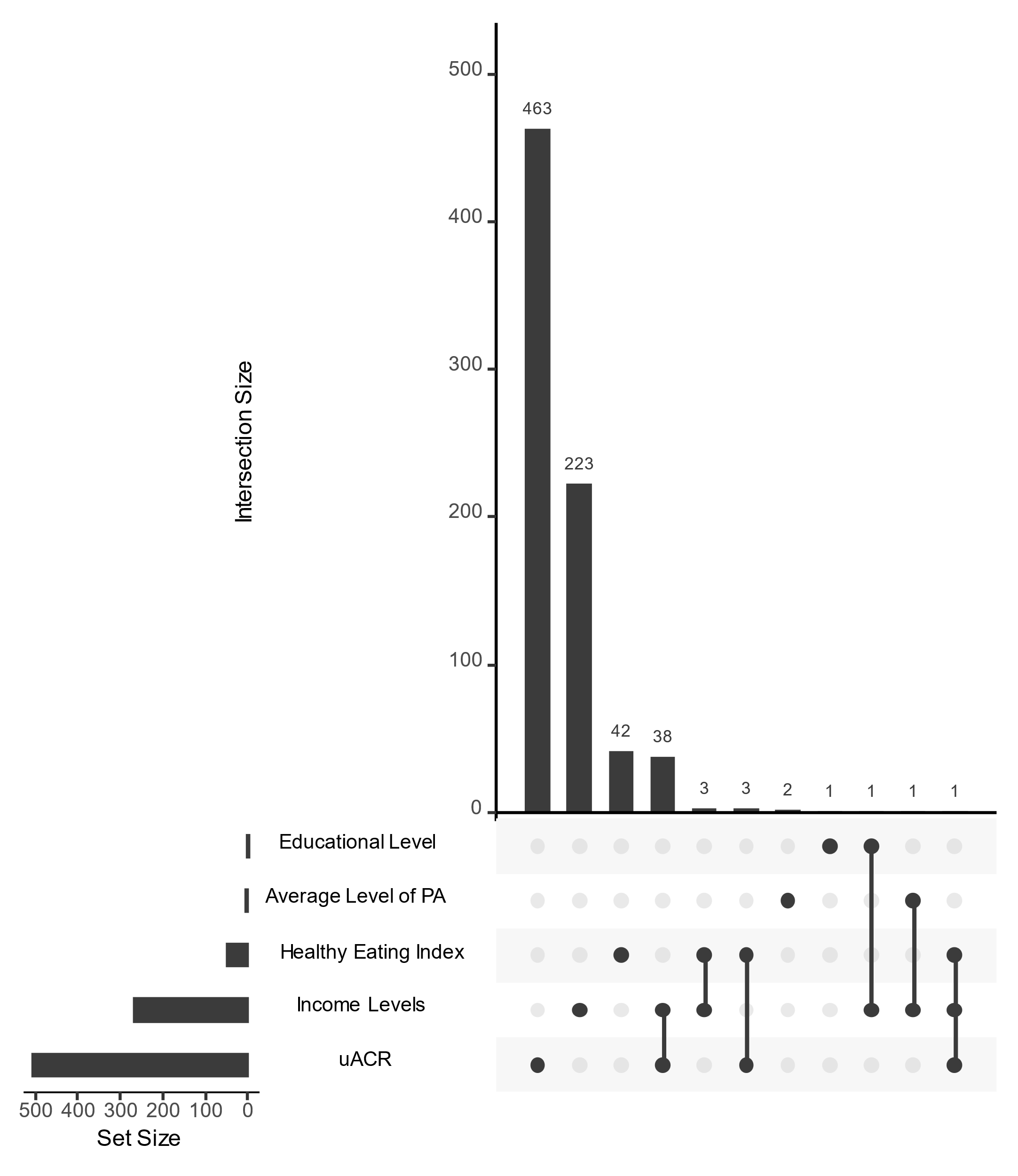


**Supplementary Figure S2.** Pattern of missing data. PA, physical activity; uACR, urine albumin-to-creatinine ratio


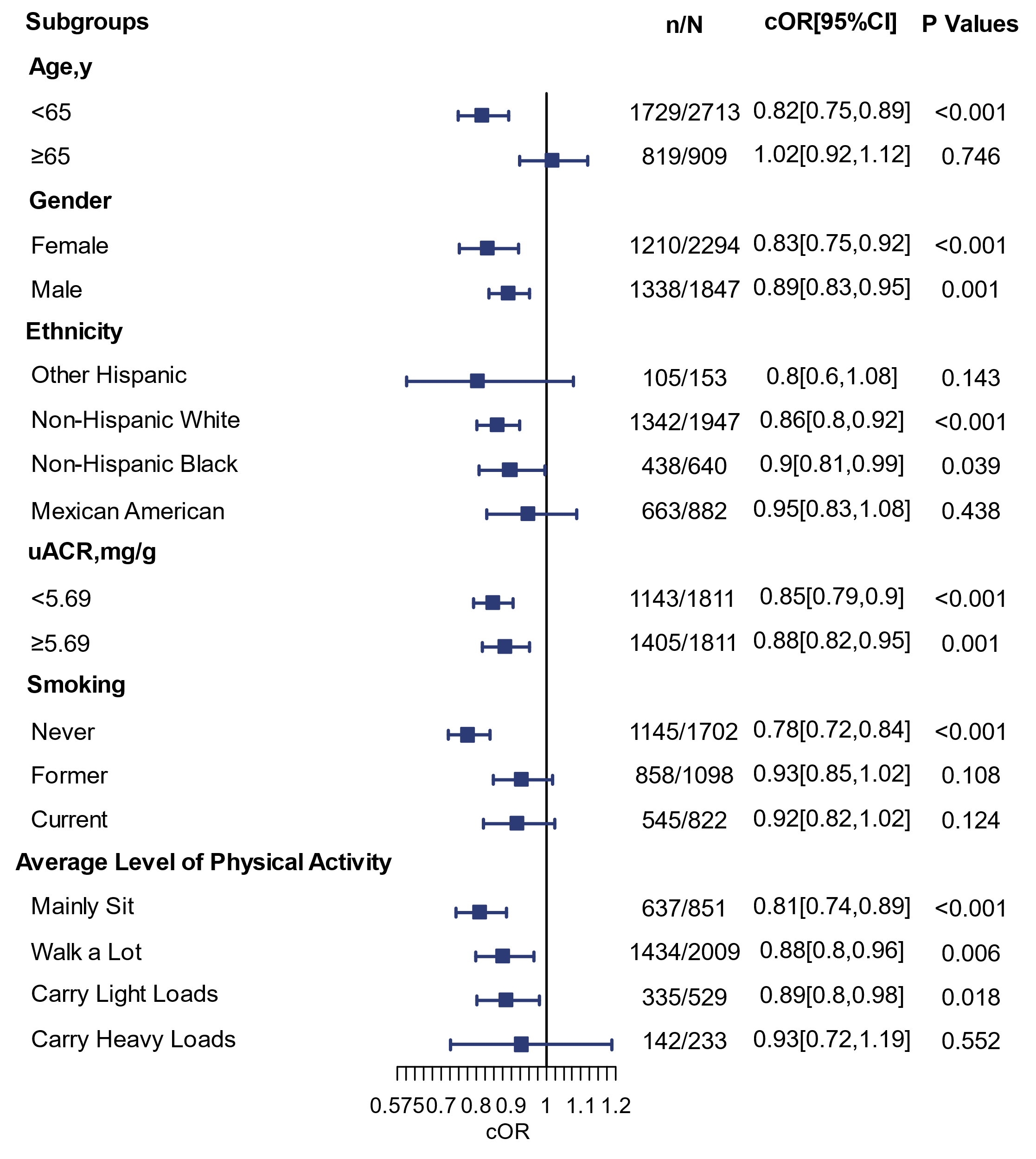


**Supplementary Figure S3.** Results of the association between eGFRdiff (treated as continuous variable) and cardiovascular-kidney-metabolic syndrome (CKM) stages in subgroups. cOR, common odd ratio; n/N, number of people in CKM stage 2-4 / total number of participants.

**Supplementary Figure S4.** Restricted cubic spine plot for the association between eGFRdiff and cardiovascular-kidney-metabolic syndrome in complete-case analysis. The restricted cubic spine model was adjusted for age, gender, ethnicity, income level, educational level, smoking status, average level of physical activity, healthy eating index, urine albumin-to-creatinine ratio and estimated glomerular filtration rate. eGFR diff; the difference between cystatin C–based estimated glomerular ﬁltration rate and creatinine based estimated glomerular ﬁltration rate.

**Supplementary Figure S5.** Restricted cubic spine plot for the association between eGFRdiff and both all-cause and cardiovascular mortality in participants with cardiovascular-kidney-metabolic (CKM) syndrome in complete-case analysis. A, all-cause mortality; B, cardiovascular mortality.

1. Hicks CW, Wang D, Matsushita K, Windham BG, Selvin E. Peripheral Neuropathy and All-Cause and Cardiovascular Mortality in U.S. Adults : A Prospective Cohort Study. Ann Intern Med. 2021;174(2):167-74.

2. NHANES 1999-2000 Questionnaire Instruments.

3. Alberti KG, Eckel RH, Grundy SM, Zimmet PZ, Cleeman JI, Donato KA, et al. Harmonizing the metabolic syndrome: a joint interim statement of the International Diabetes Federation Task Force on Epidemiology and Prevention; National Heart, Lung, and Blood Institute; American Heart Association; World Heart Federation; International Atherosclerosis Society; and International Association for the Study of Obesity. Circulation. 2009;120(16):1640-5.

4. Teklu M, Zhou W, Kapoor P, Patel N, Dey AK, Sorokin AV, et al. Metabolic syndrome and its factors are associated with noncalcified coronary burden in psoriasis: An observational cohort study. J Am Acad Dermatol. 2021;84(5):1329-38.

5. Khan SS, Matsushita K, Sang Y, Ballew SH, Grams ME, Surapaneni A, et al. Development and Validation of the American Heart Association's PREVENT Equations. Circulation. 2024;149(6):430-49.

6. Bundy JD, Mills KT, He H, LaVeist TA, Ferdinand KC, Chen J, et al. Social determinants of health and premature death among adults in the USA from 1999 to 2018: a national cohort study. Lancet Public Health. 2023;8(6):e422-e31.

7. Johnson CL, Paulose-Ram R, Ogden CL, Carroll MD, Kruszon-Moran D, Dohrmann SM, et al. National health and nutrition examination survey: analytic guidelines, 1999-2010. Vital Health Stat 2. 2013(161):1-24.

8. National Health and Nutrition Examination Survey; Physical Activity (PAQ) [Available from: <https://wwwn.cdc.gov/Nchs/Nhanes/1999-2000/PAQ.htm#PAQ180>.
